# T-cell receptor excision circle levels and safety of paediatric immunization: A population-based self-controlled case series analysis

**DOI:** 10.1080/21645515.2018.1433971

**Published:** 2018-02-26

**Authors:** Kumanan Wilson, Daniel Rodriguez Duque, Malia S.Q Murphy, Steven Hawken, Anne Pham-Huy, Jeffrey Kwong, Shelley L. Deeks, Beth K. Potter, Natasha S. Crowcroft, Dennis E. Bulman, Pranesh Chakraborty, Julian Little

**Affiliations:** a Clinical Epidemiology Program, Ottawa Hospital Research Institute, Ottawa, Canada; b School of Epidemiology and Public Health, University of Ottawa, Ottawa, Canada; c Institute for Clinical Evaluative Sciences, University of Ottawa, Ottawa, Canada; d Department of Pediatrics, University of Ottawa, Ottawa, Canada; e Dalla Lana School of Public Health, University of Toronto, Toronto, Canada; f Newborn Screening Ontario, Children's Hospital of Eastern Ontario, Ottawa, Canada

**Keywords:** adverse event following immunization, vaccine safety, pediatric immunization, pediatrics, immunization, vaccines

## Abstract

T-cell receptor excision circle levels are a surrogate marker of T-cell production and immune system function. We sought to determine whether non-pathological levels of infant T-cell receptor excision circles were associated with adverse events following immunization. A self-controlled case series design was applied on a sample of 231,693 children who completed newborn screening for severe combined immunodeficiency in Ontario, Canada between August 2013 and December 2015. Exposures included routinely administered pediatric vaccines up to 15 months of age. Main outcomes were combined health services utilization for recognized adverse events following immunization. 1,406,981 vaccination events were included in the final dataset. 103,007 children received the Pneu-C-13 or Men-C-C vaccine and 97,998 received the MMR vaccine at 12 months of age. 67,725 children received the varicella immunization at 15 months. Our analysis identified no association between newborn T-cell receptor excision circle levels and subsequent health services utilization events following DTa-IPV-Hib, Pneu-C-13, and Men-C-C vaccinations at 2-month (RI 0.94[95%CI 0.87-1.02]), 4-month (RI 0.82[95%CI 0.75-0.9]), 6-month (RI 0.63[95%CI 0.57-0.7]) and 12-month (RI 0.49[95%CI 0.44-0.55]). We also found no trends in health services utilization following MMR (RI 1.43[95%1.34-1.52]) or varicella (RI 1.14[95%CI 1.05-1.23]) vaccination. Our findings provide further support for the safety of pediatric vaccinations.

## Introduction

Pediatric vaccines have contributed to the significant reduction in the spread of infectious diseases and associated pediatric morbidity and mortality. Monitoring of vaccine safety is an active component of any immunization system, and includes clinical trials testing of new vaccines and ongoing post-market surveillance of adverse events after vaccines have been licensed for use. Although phase 3 clinical trials can typically identify adverse events occurring at a rate of 1:10,000, rarer events may not be identified, thus necessitating ongoing monitoring.^1^ The medical literature overwhelmings supports the safety of vaccination.^2^

While there is substantial evidence that vaccines are safe for healthy children,^3^^,^^4^ those with altered immune function respond differently to vaccination.^5^^,^^6^ Children with primary immunodeficiencies are at increased risk of adverse events following immunization with live-attenuated viruses or bacteria.^7–9^ In some cases, these children also have lower immunogenicity to non-live or inactivated vaccines.^10^ In the province of Ontario, data on routinely administered vaccinations are submitted through physician billing claims to the province's health insurance program, the Ontario Health Insurance Plan (OHIP). OHIP covers almost all of Ontario's approximately 13.5 million residents, except for newcomers who have resided in the province for <3 months and refugees covered under federal health programs. There is no parallel private delivery of health services in Ontario for hospitalizations. The publicly-funded Ontario immunization program^11^ offers six different vaccines for infants within the first 15 months of life: pentavalent diphtheria, pertussis, tetanus, polio, and Haemophilus influenzae type b vaccine (DTaP-IPV-HiB); 13-valent pneumococcal conjugate vaccine (Pneu-C-13); rotavirus vaccine (Rot-1); measles, mumps, and rubella (MMR) vaccine; meningococcal C (Men-C-C) vaccine; and the varicella (var) vaccine.

Severe combined immunodeficiency (SCID), a primary immunodeficiency syndrome, is the result of genetic defects that impair T-cell and B-cell development. Children with SCID are susceptible to severe infections caused by a wide range of pathogens that are not typically harmful to children with normal immunity. For this reason, vaccines with live-attenuated viruses or bacteria are contraindicated for SCID patients until a working immune system can be established.^5^^,^^6^ T-cell receptor excision circles (TRECs) are formed from mature and naïve T-cells in the course of genetic recombination^12–14^ whereby excised gene segments form stable extrachromosomal circular DNA products.^13–15^ TRECs are not replicated in the periphery during cell division, and as such they are used as surrogate markers of new T-cell production and thymic function.^13^^,^^15^ Infants with SCID have very low T-cell counts, and thus low TREC levels. TREC copy number (per microliter of blood) at birth has proven to be a sensitive and specific screening test for SCID.^16^ SCID screening has been implemented in several jurisdictions in Canada, including Ontario as well as 49 states in the United States as of May 2017.^17^ It is currently unknown whether non-pathological TREC levels in newborns are associated with vaccine reactogenicity.

Past studies have used health services utilization post-immunization as a marker of vaccine reactogenicity.^18–23^ Most live attenuated vaccines including rotavirus, measles mumps and rubella, and varicella vaccines elicit strong cell-mediated and humoral immune responses. The mechanisms through which these vaccines elicit primary T-cell activation vary by vaccine, however. We have previously demonstrated that the nature and timing of health services utilization following immunization mirrors the expected physiological response to vaccination.^18^^,^^19^ We hypothesized that differences in TREC levels at birth amongst children without SCID would predict infants' subsequent immune response and reactions to vaccines as manifested by different patterns of health services utilization following immunization.

## Results

### Baseline data

We included a total of 231,689 children and 1,406,981 vaccination events in the final dataset: 189,715 OHIP eligible children received immunizations at 2 months of age (DTap-IPV-Hib or Pneu-C-13 vaccine), 178,356 at 4 months (DTap-IPV-Hib or Pneu-C-13 vaccine), and 156,903 at 6 months (DTap-IPV-Hib vaccine only). A total of 103,007 children received the Pneu-C-13 or Men-C-C vaccine and 97,998 received the MMR vaccine at 12 months of age. 67,725 children received the varicella immunization at 15 months.

The number of health services utilization events over the course of pre- and post-vaccination periods for each vaccination timepoint are presented in Figure 3. Health services utilization events by TREC quintile for 6-month and 15-month vaccination timepoints are provided in Figure 4 to demonstrate the different service utilization trends following non-live and live attenuated vaccinations, respectively. The most frequent diagnoses associated with health services utilization events after each vaccination, by TREC quintile, are provided in Table 3.

### 2-, 4- and 6-month vaccination outcomes

189,715; 178,356; and 156,903 children received their prescribed parenteral vaccinations at 2-, 4-, and 6-months, respectively. The relative incidences of health services utilization events were 0.94(95%CI 0.87-1.02), 0.82(95%CI 0.75-0.90), and 0.63(95%CI 0.57-0.70) respectively for confirmed non-live vaccine exposure at 2-, 4-, and 6-months. We observed no statistically significant differences in RIRs across TREC quintiles for these vaccination timepoints (Table 4).

### 12-month vaccination outcomes

103,007 children received their Pneu-C-13 and Men-C-C vaccinations within 2 weeks before and 40 days after the prescribed immunization date. 367 of these children experienced one of the combined end-points within 48 hours of vaccination compared to 2,413 children in the 9-day control period. A total of 97,998 OHIP-eligible children received the MMR vaccine. Of these, 2,592 were admitted to hospital 4–12 days post-vaccination compared to 1,821 children in the 9-day control period for active vaccine exposure. The relative incidence of an event was 0.49(95%CI 0.44-0.55) and 1.43(95%CI 1.34-1.52) for non-live and live attenuated vaccine exposure, respectively. Although RIRs were lower for children with TREC levels in the second quintile compared to the highest, fifth quintile following confirmed non-live vaccination, there was no signficiant linear trend in RIRs by TREC quintile (Table 4).

### 15-month vaccination outcomes

67,725 OHIP-eligible children received a varicella immunization within 2 weeks before and 40 days after the prescribed immunization due date. Of these, 1,298 children experienced one of the combined end-points 4–12 days post-vaccination compared to 1,131 children in the 9-day control period for active vaccine exposure. The relative incidence of an event was 1.14(95%CI 1.05-1.23). The RIRs across quintiles of TREC levels did not significantly differ (Table 4).

### Sensitivity analyses

Inclusion of general vaccine billing codes did not alter the conclusions of our analyses. Comparison of patients with TREC levels in the 1^st^ and 99^th^ percentiles did not yield significantly different RIRs for any of the immunizations examined. Extending the length of the risk period for 12-month vaccinations did not identify an effect for any of the analyses. No effect was identified by adding in a live risk period for the 2- and 4-month immunizations. Examination of all-cause ED visits and hospitalizations yielded the same conclusions as the main analysis. (Appendices C-F)

## Discussion

Our analysis demonstrated that newborn TREC levels were not associated with increased health services utilization post-immunization for vaccines received at 2-, 4-, 6-, 12- and 15- months of age. To our knowledge, this is the first study to examine measures of immunity at birth and health services utilization following immunization.

For an effective immune response to immunization, both the innate and adaptive immune systems need to be activated. Depending on their mechanism of action, vaccines invoke a direct influence on immune effectors. Many inactivated vaccines are extracellular antigens and they induce mainly antibody responses. In contrast, attenuated live vaccines deliver anitgens to the interior of antigen presenting cells to elicit strong T-cell-mediated immunity. Immune responses elicited by live attenuated vaccines are similar to those occurring after a natural infection. Therefore, they efficiently trigger the activation of both the innate and humoral immune systems and usually generate a stronger CD8+ cytotoxic T-cell response.^24^^,^^25^ Our findings are reassuring as they provide further evidence to support the safety of routine pediatric immunizations.

Strengths of our study include the direct linkage between newborn screening and health services data, the population-based nature of the analysis and the use of the SCCS design. Using this system, we have conducted several analyses of vaccine safety.^18^^,^^19^^,^^26–28^ Spikes in health services utilization in the time following vaccination may constitute a vaccine safety signal and serve as a measure of vaccine reactogenicity. Exploration of the reasons for health services utilization allows for signal validation and hypothesis generation. Use of vaccine specific codes with demonstrated high specificity and moderate sensitivity in pediatric patients^29^ additionally ensured that subjects were appropriately identified based on exposure status. Another strength of our approach is our use of relative incidence ratios. As a result of the healthy vaccinee effect (*i.e.* children are vaccinated at a time when they are particularly healthy and unwell children often defer vaccination),^30–32^ risk of health services utilization immediately post-vaccination is often lower than overall baseline risks pre-vaccination. Relative incidence ratios enabled us to compare this risk across groups to uncover interactions that may otherwise have beeen masked.

Our study also had limitations. First, our main analysis was limited to vaccines with specific billing codes. While we restricted our analysis to examination of the non-live DTaP-IPV-HiB vaccine at 2, 4, and 6 months of age, children receiving this vaccine may also have received the oral live-attenuated rotavirus vaccine at 2 and 4 months, for which there is no specific code. Sensitivity analyses to address this found no effect, however. Second, our study is potentially limited by our use of TRECs as the primary measure of immune system function. The use of TREC as a screening test for SCID has been shown to be nearly 100% sensitive for the identification of classic SCID patients. Collective data from multiple jurisdictions that have implemented SCID screening have also shown that the TREC assay also detects a variety of genetic and syndromic T-cell lymphopenia disorders, as well as secondary causes of T-cell lymphopenia.^33^ Although TRECs have been used to examine immune system function across an array of conditions, it is unclear whether newborn TREC levels may be used as a long-term measure of immunity, and it has not been established whether TREC levels at birth are correlated with levels at the time of vaccination or correlate with immunity in healthy children. In addition to the detection of neonatal primary immunodeficiency, TRECs have been used to examine success of antiretroviral therapy^15^^,^^34^^,^^35^ and bone marrow transplantation.^36–39^

This study represents further exploration in the emerging field of examining predictors of adverse events to immunization using biomarkers.^40–43^ Large databases of genetic, proteomic, immunogenic and metabolic markers can be combined with novel bostatistical approaches can be utilized to identify individuals who may be susceptible to adverse events from immunization. Future work related to this study should seek to determine if TREC levels change over the course of childhood and, in particular, whether TREC levels at the time of newborn screening are correlated with levels at the time of subsequent immunization. Comparison of TREC levels of children with and without recognized adverse events following immunization is also warranted as well as the general correlation of TREC levels in non SCID infants and subsequent health outcomes. Our analyses suggest, however, that newborn TREC levels do not predict health services utilization following childhood immunization and provide further support for the safety of routine 2-, 4-, 6-, 12-, and 15-month vaccinations amongst the pediatric population.

## Methods

### Study design

**Figure 1.**
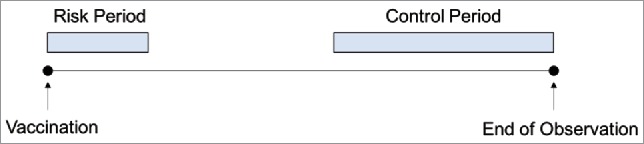
Self-Controlled Case-Series Design. The observation period for each patient begins with vaccination. The risk period denotes a time when the likelihood of an outcome (ED visit, or hospital admission) related to the vaccination is most likely. The control period captures a window of time when the likelihood of an outcome related to vaccination is unlikely. The intervening days represent a ‘wash-out’ period.

We used the self-controlled case series (SCCS) design.^20^ The SCCS methodology relies on exposed cases that have experienced the primary outcome of interest, the incidence rate of health services events (emergency department, ED, visit or hospital admission). In our study, children contributed towards SCCS estimates if they had both received a vaccine and had at least one health service event during the study period. The SCCS is frequently used for evaluating vaccine safety.^44–51^ SCCS uses conditional Poisson modelling to compare the incidence rate of health services utilization in the post-vaccination (risk) period to the incidence during the unexposed (control) period, during which it would be unlikely for the vaccination affect the outcome of interest (Figure 1). Comparing incidence rates as opposed to number of events, eliminates issues that may arise when comparing risk and control periods of differing lengths. The conditional component of the model arises from the fact that the SCCS method allows for person-specific effects on the incidence rate, however these were not of primary interest to the study. It was therefore possible to estimate parameters of interest without estimating extra nuisance parameters. As a self-controlled analysis, this approach implicitly adjusts for all measured and unmeasured time-invariant covariates.^15^^,^^34^ A survey of the SCCS methodology is presented by Whitaker et al.^47^

### Participants

This study included 231,689 children who completed newborn screening for SCID in Ontario between its implementation on August 12^th^ 2013 and December 31^st^ 2015 and who received at least one of the provincially recommended pediatric vaccines.

### Exposures

Exposures of interest were routinely administered pediatric vaccines up to 15 months of age for which administrative data exist. The Ontario Routine Pediatric Immunization Schedule for children 0–15 months of age is provided in Table 1. Of these, the rotavirus, varicella and MMR vaccines are live attenuated virus vaccines and the remainder are non-live. All vaccines with the exception of rotavirus vaccine are given parenterally; rotavirus vaccine is given orally. Vaccine-specific billing codes are available for all of the recommended parenteral pediatric vaccines. These codes have previously been validated by Shwartz et al. in a pediatric setting and have been shown to have specificity ranging from 88.5% – 91.5% and sensitivity ranging from 70.1-72.4%.^29^ All vaccines for which vaccine-specific billing codes were available for this age group were examined.

**Table 1.** Ontario Routine Pediatric Immunization Schedule for children 0–15 months of age.

Vaccine	2-months	4-months	6-months	12-months	15-months
DTap-IPV-Hib	**•**	**•**	**•**		
Pneu-C-13	**•**	**•**		**•**	
Rot-1	**♦**	**♦**			
Men-C-C				**•**	
MMR				**•**	
Var					**•**

♦no vaccine-specific code available; •vaccine-specific codes available

### Outcomes

Our outcome of interest was the incidence rate for combined health services utilization, specifically ED visits and hospitalizations, for recognized adverse events following immunization. Events not likely associated with the vaccination itself were excluded (Appendix A). Where multiple events occurred on the same day (e.g. an ED visit leading to a hospital admission) we counted only one event.

### Data sources

Provincial newborn screening data and health service data, including vaccine administration, are available as linked datasets housed at the Institute for Clinical Evaluative Sciences (ICES; Toronto, Canada). All datasets were linked using unique encoded identifiers. The Newborn Screening Ontario (NSO) dataset was used to obtain TREC screening levels and other relevant birth data. Approximately 140,000 children are born each year in Ontario, virtually all (>99%) of whom undergo newborn screening. Initial data cleaning removed duplicate records, mismatched or invalid mother-infant OHIP numbers, ‘unsatisfactory’ newborn screening samples, and incomplete records (for those infants with multiple screening records on file).

**Figure 2.**
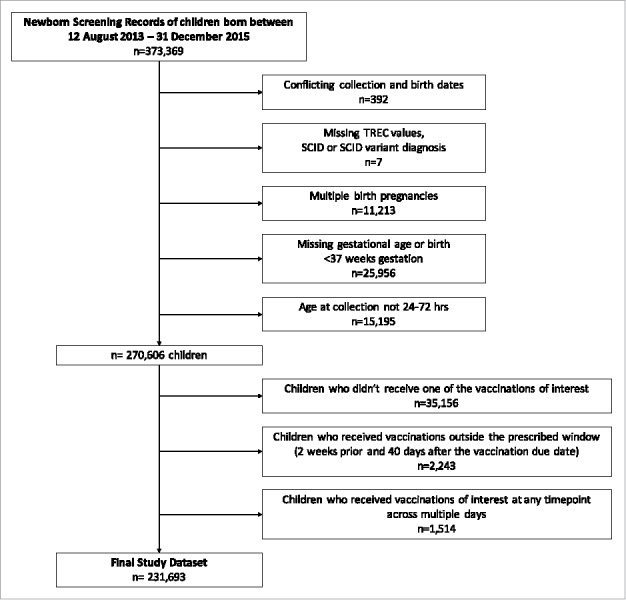
Selection of infants from Newborn Screening Ontario dataset.

Data were further cleaned to remove those records with missing or conflicting birth or collections dates (data entry errors), TREC values, and gestational ages. Infants who received a definitive diagnosis of SCID or SCID variants were excluded from the analysis, as were multi-fetal births, preterm births (birth at <37 weeks gestational age) and infants who were tested outside of the prescribed 24–72 hours sampling period, as TREC levels in these infant groups may be unreliable due to a variety of physiological sequelae. A flowchart depicting cohort creation is provided in Figure 2.

**Figure:**
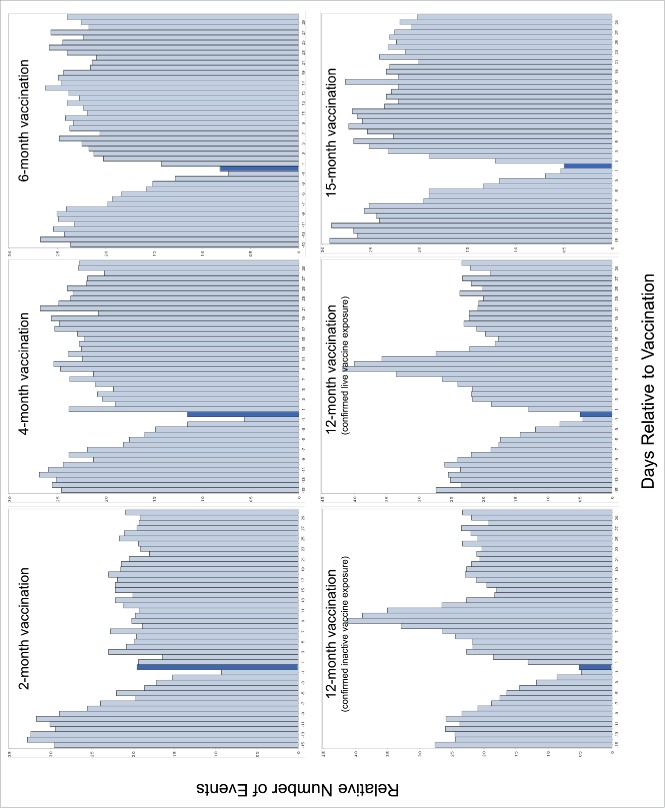

**Figure:**
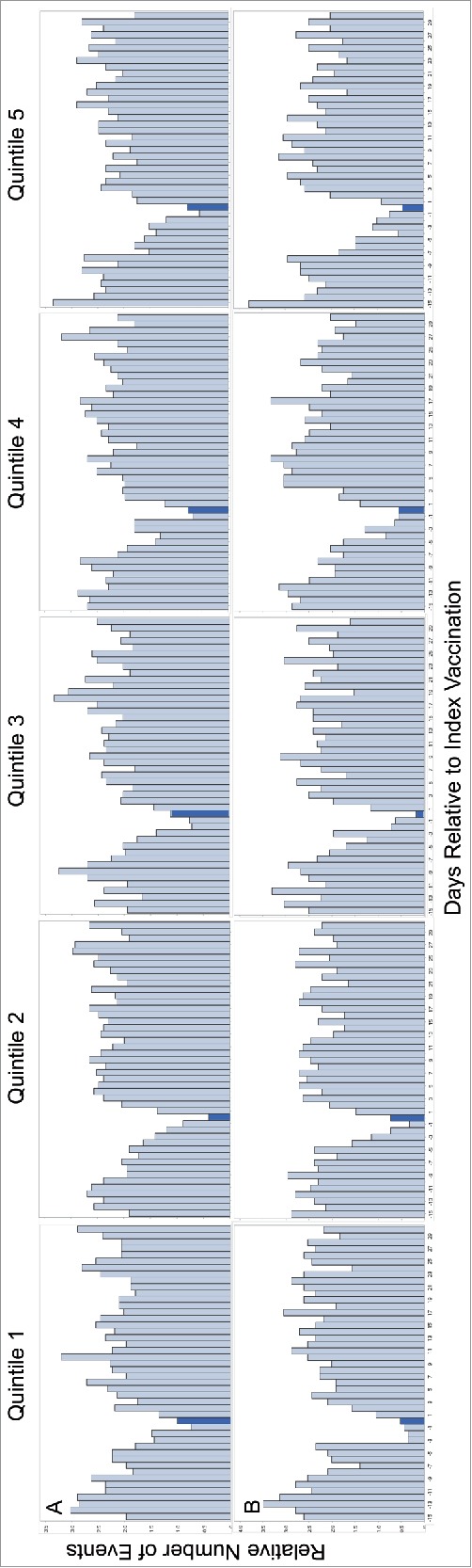

Pediatric vaccinations were identified using vaccine-specific billing codes available from the OHIP claims database. The Canadian Institute for Health Information's (CIHI) Discharge Abstract Database was used to identify all hospital admissions in the province, and CIHI's National Ambulatory Care Reporting System was used to identify ED visits. Lastly, the Registered Persons Database was used to ascertain OHIP coverage eligibility and date of death, if applicable.

### Statistical methods

We examined the association between quintile of TREC levels and health services utilization. TREC levels were standardized by calendar week of sample collection and by patient age at the time of collection. The resulting standardized series permitted exploration of whether relatively low or high TREC values differed with respect to the primary outcomes of interest, within each quintile. Details of our statistical analyses are provided in the appendix.

At each timepoint, infants were included if there was record of the infant receiving at least one of the provincially recommended vaccines. Infants were included for 2-, 4-, 6-month vaccination analyses if they were eligible for OHIP coverage at 6 months of age. Infants were included for 12- or 15-month vaccination analyses if they were eligble for OHIP at 12- or 15-months of age respectively. Each vaccination point was analysed (modelled) independently from the others. Vaccinations were confirmed through examination of vaccine-specific billing codes (Appendix B) and were included if they were administered up to 14 days before and 40 days after the vaccination due date. Children were excluded if they didn't receive one of the vaccinations of interest, received vaccinations outside of the prescribed window of vaccination, or received vaccinations on multiple days within a given vaccination window were not included in the analysis.

We examined health services events occurring during pre-specified risk and control periods relative to immunization. Risk and control periods were defined *a priori* based on expected timing and character of reactogenicity to vaccination^26^^,^^52^ (Table 2). Risk periods were based on the biologically expected impact of the vaccine and our previous work.^18^^,^^28^^,^^53^ 0–48 hours post-immunization was used as the risk period for non-live vaccines, and 4–12 days post-immunization for live-attenuated vaccines. The control periods were based on when the number of events post-vaccination had equilibrated, as determined through graphical examination. We elected not to use a pre-vaccine control period as we have previously demonstrated that the number of hospital events steadily drop after birth. Using a pre-vaccine control period would have therefore biased against detecting a signal. Post-immunization events following exposure to live attenuated or non-live vaccinations were independently examined for the 12-month months-of-age timepoint at which both live attenuated and non-live vaccines are recommended. Our work has previously demonstrated that these two risk intervals are sufficiently distinct and there is unlikely to be contamination of events between these risk intervals.^18^ For each vaccination point, relative incidences were presented comparing risk and control period using an SCCS model containing only the period effect.

**Table 2.** Risk and control periods by exposure type and vaccination.

Exposure Type	Vaccination Timepoints	Risk Period	Control Period
Confirmed non-live vaccines	2,-,4-,6- and 12-month	0-48 hours post-vaccination	9-18 days post-vaccination
Confirmed live attenuated vaccines	12- and 15-month	4-12 days post-vaccination	20-28 days post-vaccination

**Table 3.** Top 3 diagnoses associated with health services utilization by vaccination period.

Vaccination Period	Top 3 diagnoses
2-month	Fever, unspecified; Acute upper respiratory infection, unspecified; Other complications following immunization, not elsewhere classified
4-month	Fever, unspecified; Acute upper respiratory infection, unspecified; Viral infection, unspecified
6-month	Vomiting alone; Acute upper respiratory infection, unspecified; Fever, unspecified
12-month non-live	Fever, unspecified; Acute upper respiratory infection, unspecified; Viral infection, unspecified
12-month active	Acute upper respiratory infection, unspecified; Otitis media, unspecified; Fever, unspecified;
15-month	Acute upper respiratory infection, unspecified; Otitis media, unspecified; Fever, unspecified;

**Table 4.** RIRs for combined ED and inpatient visits following vaccine administration.

Confirmed non-live vaccine administration							
	TREC Quintile	Vaccinated Children, n (%)	Events During Risk Period (Days 0–2), n	Events During Control Period (Days 9–18), n	Relative Incidence (95% CI)	RIR (95% CI)	RIR p-value
*2-month (DTap-IPV-Hib; Pneu-C-13)*	Overall	189715	827	2820	0.94(0.87-1.02)		
	1	37494 (19.8)	152	592	0.84(0.69-1.01)	0.87(0.67-1.12)	0.52
	2	37827 (19.9)	172	569	0.99(0.82-1.18)	1.02(0.79-1.31)	
	3	38108 (20.1)	154	554	0.89(0.73-1.07)	0.92(0.71-1.19)	
	4	38229 (20.2)	179	553	1.03(0.86-1.23)	1.06(0.82-1.37)	
	5	38057 (20.1)	170	552	0.97(0.81-1.16)	Reference	
*4-month (DTap-IPV-Hib; Pneu-C-13)*	Overall	178356	623	2432	0.82(0.75-0.9)		
	1	34802 (19.5)	136	491	0.88(0.72-1.08)	1.13(0.84-1.51)	0.32
	2	35740 (20)	142	496	0.94(0.77-1.14)	1.2(0.9-1.6)	
	3	36157 (20.3)	117	469	0.82(0.66-1.01)	1.05(0.78-1.41)	
	4	35923 (20.1)	115	509	0.7(0.57-0.87)	0.9(0.67-1.22)	
	5	35734 (20)	113	467	0.78(0.63-0.97)	Reference	
*6-month (DTap-IPV-Hib)*	Overall	156903	476	2381	0.63(0.57-0.7)		
	1	30728 (19.6)	104	485	0.68(0.54-0.84)	1(0.73-1.37)	0.64
	2	31503 (20.1)	86	487	0.57(0.45-0.72)	0.84(0.6-1.17)	
	3	31760 (20.2)	103	478	0.67(0.53-0.84)	1(0.72-1.37)	
	4	31532 (20.1)	88	482	0.57(0.45-0.73)	0.85(0.61-1.18)	
	5	31380 (20)	95	449	0.67(0.53-0.85)	Reference	
*12-month (Pneu-C-13; Men-C-C)*	Overall	103007	367	2413	0.49(0.44-0.55)		0.25
	1	19596 (19)	59	443	0.43(0.32-0.57)	0.72(0.5-1.04)	
	2	21433 (20.8)	68	517	0.42(0.32-0.54)	0.69(0.48-0.99)	
	3	21078 (20.5)	80	517	0.5(0.39-0.64)	0.84(0.59-1.18)	
	4	20702 (20.1)	75	482	0.52(0.4-0.67)	0.86(0.61-1.22)	
	5	20198 (19.6)	85	454	0.6(0.47-0.76)	Reference	
Confirmed Live attenuated Vaccine administration							
	TREC Quintile	Vaccinated Children, n	Events During Risk Period (Days 4–12), n	Events During Control Period (Days 20–28), n	Relative Incidence (95% CI)	RIR (95% CI)	RIR p-value
*12-month (MMR)*	Overall	97998	2592	1821	1.43(1.34-1.52)		
	1	18664 (19)	499	298	1.62(1.39-1.88)	1.15(0.93-1.41)	0.10
	2	20391 (20.8)	558	369	1.51(1.31-1.74)	1.07(0.87-1.31)	
	3	20082 (20.5)	515	411	1.24(1.08-1.42)	0.88(0.72-1.07)	
	4	19635 (20)	511	372	1.42(1.23-1.64)	1.01(0.82-1.23)	
	5	19226 (19.6)	509	371	1.41(1.22-1.63)	Reference	
*15-month (Var)*	Overall	67725	1298	1131	1.14(1.05-1.23)		
	1	12817 (18.9)	238	252	0.97(0.81-1.18)	0.82(0.62-1.07)	0.13
	2	14228 (21)	277	239	1.12(0.93-1.35)	0.94(0.72-1.23)	
	3	13909 (20.5)	240	230	1.07(0.88-1.3)	0.9(0.68-1.18)	
	4	13638 (20.1)	282	202	1.37(1.13-1.65)	1.15(0.87-1.5)	
	5	13133 (19.4)	261	208	1.19(0.98-1.45)	Reference	

For each vaccination point, we additionally explored the relationship between infant TREC levels and incidence rates during the risk and control periods, TREC level groupings were based on the population level quintiles, after exclusion criteria were applied. To explore this, we used the SCCS model with a period main effect and a quintile by period interaction. In the same way as Hawken et al, we calculated relative incidence ratios (RIRs) for each TREC quintile versus the highest TREC quintile, designated as the reference category.^53^ The RIR estimate represents the change in relative incidence between the risk and control period for each TREC quintile relative to the reference quintile. P-values of the likelihood ratio test for interaction in the SCCS model were used to establish whether the RIR for TREC level was statistically significant.^47^

We conducted five sensitivity analyses to ensure the conclusions reached were robust. (1) We re-ran analyses to include general vaccine codes. (2) We separated the 1^st^ and 99^th^ percentiles of TREC levels to determine whether extreme values had a differential relative rate of adverse events compared to the reference percentile (80^th^-99^th^). (3) For 12-month vaccinations, we extended the live-vaccine risk period to 0–12 days after vaccination to allow for the possibility of early-occurring events. (4) We applied live-vaccine risk period analyses at 2 and 4 months to account for individuals who may have receieved the rotavirus vaccine, which is not captured by billing codes. (5) Instead of considering restricted event outcomes (excluding diagnoses clearly unrelated to vaccination), we included any ED or hospitalization. We conducted all analyses in SAS version 9.3 (SAS Institute, Cary, NC) and uses SAS macros for fitting the SCCS series (http://statistics.open.ac.uk/sccs).

## Abbreviations

CIHI: Canadian Institute for Health Information
DNA: deoxyribonucleic acid
DTaP-IPV-HiB: diphtheria, pertussis, tetanus, polio, and Haemophilus influenzae type b vaccine
ED: emergency department
ICES: Institute for Clinical Evaluative Sciences
MMR: measles, mumps, and rubella vaccine
Men-C-C: meningococcal C vaccine
MOHLTC: Ministry of Health and Long Term Care
OHIP: Ontario health insurance plan
Pneu-C-13: 13-valent pneumococcal conjugate vaccine
RIR: relative incidence ratio
Rot-1: rotavirus vaccine
SCID: severe combined immunodeficiency
SCCS: self-controlled case series
TREC: T-cell receptor excision circle
Var: varicella

## Data statement

The data used in this study are available with permission from the Institute for Clinical Evaluative Sciences.

## Other

There are no prior publications or submissions with any overlapping information, including studies and patients.

## Appendix A. Codes excluded for the restricted outcomes

**Table:**

Code	Description
E00-E90	Endocrine, nutritional and metabolic diseases
F00-F99	Mental and behavioural disorders
K4	Inguinal hernia
Q00-Q99	Congenital Anomalies
S	Injury, poisoning and certain other consequences of external causes (S00-T98)
T0	Superficial injuries involving multiple body regions Open wounds involving multiple body regions Fractures involving multiple body regions Dislocations, sprains and strains involving multiple body regions Crushing injuries involving multiple body regions Traumatic amputations involving multiple body regions Other injuries involving multiple body regions, not elsewhere classified Unspecified multiple injuries Fracture of spine, level unspecified Other injuries of spine and trunk, level unspecified
T1	Fracture of upper limb, level unspecified Other injuries of upper limb, level unspecified Fracture of lower limb, level unspecified Other injuries of lower limb, level unspecified Injury of unspecified body region Foreign body on external eye Foreign body in respiratory tract Foreign body in alimentary tract Foreign body in genitourinary tract
T2	Burn and corrosion of head and neck Burn and corrosion of trunk Burn and corrosion of shoulder and upper limb, except wrist and hand Burn and corrosion of wrist and hand Burn and corrosion of hip and lower limb, except ankle and foot Burn and corrosion of ankle and foot Burn and corrosion confined to eye and adnexa Burn and corrosion of respiratory tract Burn and corrosion of other internal organs Burns and corrosions of multiple body regions
T30	Burn and corrosion, body region unspecified
T31	Burns classified according to extent of body surface involved
T32	Corrosions classified according to extent of body surface involved
T33	Superficial frostbite
T34	Frostbite with tissue necrosis
T35	Frostbite involving multiple body regions and unspecified frostbite
T79	Certain early complications of trauma, not elsewhere classified
C, D0-D4	Neoplasm

## Appendix B. Vaccine Specific Codes

**Table:**

Vaccination	Code
DTaP-IPV-HiB	G840,G841,G847
Pneu-C-13	G846
Men-C-C	G844
MMR	G845
Varicella	G848

## Appendix C. Sensitivity Analysis – Inclusion of General Vaccine Billing Codes

**Table:**

Confirmed non-live Vaccine Exposure							
	TREC Quintile	Vaccinated Children, n(%)	Risk Period Events (Days 0-2), n	Control Period Events (Days 9-18), n	Relative Incidence (95% CI)	RIR (95% CI)	RIR p-value
*2-month*	Overall	200834	876	3006	0.94(0.87-1.02)		
	1	39742 (19.8)	161	632	0.83(0.69-1)	0.84(0.65-1.08)	0.41
	2	40055 (19.9)	181	606	0.98(0.82-1.16)	0.98(0.77-1.26)	
	3	40328 (20.1)	159	585	0.87(0.72-1.05)	0.88(0.68-1.13)	
	4	40477 (20.2)	191	593	1.02(0.86-1.22)	1.03(0.8-1.31)	
	5	40232 (20)	184	590	0.99(0.83-1.18)	Reference	
*4-month*	Overall	188531	667	2581	0.83(0.76-0.9)		
	1	36808 (19.5)	145	524	0.87(0.72-1.06)	1.12(0.84-1.48)	0.30
	2	37791 (20)	151	522	0.95(0.78-1.15)	1.21(0.92-1.6)	
	3	38176 (20.2)	128	500	0.84(0.68-1.02)	1.07(0.8-1.42)	
	4	37998 (20.2)	123	541	0.71(0.58-0.87)	0.91(0.68-1.21)	
	5	37758 (20)	120	494	0.78(0.63-0.96)	Reference	
*6-month*	Overall	166792	509	2560	0.63(0.57-0.69)		
	1	32667 (19.6)	109	520	0.66(0.53-0.82)	0.98(0.72-1.33)	0.43
	2	33507 (20.1)	91	522	0.55(0.43-0.69)	0.82(0.59-1.12)	
	3	33754 (20.2)	114	514	0.7(0.56-0.87)	1.04(0.77-1.42)	
	4	33537 (20.1)	93	516	0.57(0.45-0.71)	0.84(0.61-1.16)	
	5	33327 (20)	102	488	0.67(0.53-0.84)	Reference	
*12-month)*	Overall	111653	401	2619	0.49(0.44-0.55)		0.32
	1	21278 (19.1)	65	483	0.43(0.33-0.57)	0.72(0.51-1.02)	
	2	23258 (20.8)	80	557	0.44(0.34-0.57)	0.74(0.52-1.03)	
	3	22862 (20.5)	84	560	0.48(0.38-0.62)	0.8(0.58-1.12)	
	4	22411 (20.1)	78	526	0.49(0.38-0.63)	0.82(0.58-1.14)	
	5	21844 (19.6)	94	493	0.60(0.48-0.76)	Reference	
Confirmed Live attenuated Vaccine Exposure							
	TREC Quintile	Vaccinated Children, n(%)	Risk Period Events (Days 4-12), n	Control Period Events (Days 20-28), n	Relative Incidence (95% CI)	RIR (95% CI)	RIR p-value
*12-month*	Overall	112341	2937	2110	1.4(1.32-1.48)		0.20
	1	21407 (19.1)	556	354	1.53(1.33-1.76)	1.13(0.93-1.38)	
	2	23415 (20.8)	628	426	1.48(1.29-1.69)	1.09(0.9-1.32)	
	3	22985 (20.5)	588	467	1.24(1.09-1.42)	0.92(0.76-1.11)	
	4	22560 (20.1)	596	432	1.42(1.24-1.63)	1.05(0.87-1.27)	
	5	21974 (19.6)	569	431	1.35(1.18-1.54)	Reference	
*15-month*	Overall	72885	1382	1221	1.12(1.03-1.21)		0.19
	1	13810 (18.9)	258	268	1(0.83-1.19)	0.87(0.67-1.13)	
	2	15345 (21.1)	297	260	1.11(0.92-1.32)	0.97(0.75-1.25)	
	3	14976 (20.5)	251	246	1.04(0.87-1.26)	0.91(0.7-1.19)	
	4	14634 (20.1)	299	216	1.34(1.11-1.61)	1.17(0.9-1.52)	
	5	14120 (19.4)	277	231	1.14(0.95-1.38)	Reference	

## Appendix D. Sensitivity Analysis - SCCS Model separating top and bottom 1% of TREC values

**Table:**

Confirmed non-live Vaccine Exposure							
	TREC Quintile	Vaccinated Children, n(%)	Risk Period Events (Days 0-2), n	Control Period Events (Days 9-18), n	Relative Incidence (95% CI)	RIR (95% CI)	RIR p-value
2-month	Overall	189715	827	2820	0.94(0.87-1.02)		
	0-1%	1792 (0.9)	11	33	1.04(0.48-2.22)	1.07(0.48-2.34)	0.74
	1%-20%	35702 (18.8)	141	559	0.83(0.68-1.01)	0.85(0.65-1.11)	
	20%-40%	37827 (19.9)	172	569	0.99(0.82-1.18)	1.02(0.78-1.32)	
	40%-60%	38107 (20.1)	154	554	0.89(0.73-1.07)	0.91(0.7-1.19)	
	60%-80%	38229 (20.2)	179	553	1.03(0.86-1.23)	1.06(0.81-1.37)	
	80%-99%	36185 (19.1)	158	510	0.97(0.8-1.17)	Reference	
	99%-100%	1873 (1)	12	42	0.92(0.46-1.81)	0.94(0.46-1.9)	
4-month	Overall	178356	623	2432	0.82(0.75-0.9)		
	0-1%	1552 (0.9)	11	30	1.04(0.48-2.22)	1.3(0.59-2.86)	0.45
	1%-20%	33250 (18.6)	125	461	0.87(0.71-1.07)	1.09(0.81-1.47)	
	20%-40%	35740 (20)	142	496	0.94(0.77-1.14)	1.18(0.88-1.57)	
	40%-60%	36156 (20.3)	117	469	0.82(0.66-1.01)	1.02(0.76-1.38)	
	60%-80%	35923 (20.1)	115	509	0.7(0.57-0.87)	0.88(0.65-1.19)	
	80%-99%	33952 (19)	109	439	0.8(0.64-0.99)	Reference	
	99%-100%	1783 (1)	4	28	0.48(0.16-1.38)	0.6(0.2-1.77)	
6-month	Overall	156903	476	2381	0.63(0.57-0.7)		
	0-1%	1300 (0.8)	8	24	1.14(0.5-2.59)	1.69(0.72-3.96)	0.64
	1%-20%	29428 (18.8)	96	461	0.65(0.52-0.82)	0.97(0.7-1.34)	
	20%-40%	31503 (20.1)	86	487	0.57(0.45-0.72)	0.84(0.6-1.17)	
	40%-60%	31759 (20.2)	103	478	0.67(0.53-0.84)	1(0.72-1.38)	
	60%-80%	31532 (20.1)	88	482	0.57(0.45-0.73)	0.85(0.61-1.18)	
	80%-99%	29822 (19)	90	427	0.67(0.53-0.86)	Reference	
	99%-100%	1559 (1)	5	22	0.68(0.25-1.81)	1.01(0.37-2.75)	
12-month	Overall	103007	367	2413	0.49(0.44-0.55)		
	0-1%	750 (0.7)	2	21	0.29(0.06-1.22)	0.48(0.11-2.09)	0.46
	1%-20%	18846 (18.3)	57	422	0.44(0.33-0.58)	0.73(0.5-1.07)	
	20%-40%	21433 (20.8)	68	517	0.42(0.32-0.54)	0.7(0.48-0.99)	
	40%-60%	21077 (20.5)	80	517	0.5(0.39-0.64)	0.84(0.59-1.18)	
	60%-80%	20703 (20.1)	75	482	0.52(0.4-0.67)	0.86(0.61-1.23)	
	80%-99%	19179 (18.6)	81	435	0.6(0.46-0.77)	Reference	
	99%-100%	1019 (1)	4	19	0.63(0.21-1.86)	1.06(0.35-3.2)	
Confirmed Live attenuated Vaccine Exposure							
Timepoint	TREC Quintile	Vaccinated Children, n(%)	Risk Period Events (Days 4-12), n	Control Period Events (Days 20-28), n	Relative Incidence (95% CI)	RIR (95% CI)	RIR p-value
12-month	Overall	97998	2592	1821	1.43(1.34-1.52)		
	0-1%	713 (0.7)	20	13	1.5(0.72-3.12)	1.08(0.51-2.28)	0.19
	1%-20%	17951 (18.3)	479	285	1.63(1.39-1.9)	1.17(0.94-1.45)	
	20%-40%	20391 (20.8)	558	369	1.51(1.31-1.74)	1.09(0.88-1.33)	
	40%-60%	20081 (20.5)	515	411	1.24(1.08-1.42)	0.89(0.73-1.09)	
	60%-80%	19636 (20)	511	372	1.42(1.23-1.64)	1.02(0.83-1.25)	
	80%-99%	18222 (18.6)	486	359	1.39(1.2-1.61)	Reference	
	99%-100%	1004 (1)	23	12	2(0.96-4.13)	1.44(0.68-3.01)	
15-month	Overall	67725	1298	1131	1.14(1.05-1.23)		
	0-1%	438 (0.6)	10	11	1(0.41-2.41)	0.83(0.33-2.04)	0.28
	1%-20%	12379 (18.3)	228	241	0.97(0.8-1.18)	0.8(0.61-1.06)	
	20%-40%	14228 (21)	277	239	1.12(0.93-1.35)	0.93(0.7-1.21)	
	40%-60%	13908 (20.5)	240	230	1.07(0.88-1.3)	0.89(0.67-1.17)	
	60%-80%	13639 (20.1)	282	202	1.37(1.13-1.65)	1.13(0.86-1.49)	
	80%-99%	12467 (18.4)	251	197	1.21(0.99-1.47)	Reference	
	99%-100%	666 (1)	10	11	0.9(0.36-2.22)	0.74(0.29-1.88)	

## Appendix E. Sensitivity Analysis –RIR for extended risk period (0-12 days) for individuals with confirmed live attenuated vaccine exposure

**Table:**

Timepoint	TREC Quintile	Vaccinated Children	Risk Period Events (Days 0-12)	Control Period Events (Days 20-28)	Relative Incidence (95% CI)	RIR (95% CI)	RIR p-value
12-month	Overall	97998	3146	1821	1.18(1.11-1.26)		
	0-1%	713 (0.7)	23	13	1.15(0.56-2.37)	0.97(0.46-2.02)	0.2415
	1%-20%	17951 (18.3)	569	285	1.32(1.13-1.54)	1.11(0.91-1.37)	
	20%-40%	20391 (20.8)	664	369	1.24(1.08-1.42)	1.04(0.86-1.27)	
	40%-60%	20081 (20.5)	630	411	1.04(0.91-1.19)	0.88(0.72-1.07)	
	60%-80%	19636 (20)	620	372	1.16(1-1.33)	0.98(0.8-1.19)	
	80%-99%	18222 (18.6)	609	359	1.19(1.03-1.37)	Reference	
	99%-100%	1004 (1.0)	31	12	1.82(0.91-3.65)	1.54(0.75-3.13)	
15-month	Overall	67725	1632	1131	0.97(0.9-1.05)		
	0-1%	941 (1.4)	13	11	0.9(0.39-2.06)	0.88(0.37-2.06)	0.2894
	1%-20%	13778 (20.3)	285	241	0.82(0.68-0.98)	0.8(0.61-1.04)	
	20%-40%	12926 (19.1)	361	239	0.99(0.83-1.19)	0.97(0.75-1.26)	
	40%-60%	12012 (17.7)	305	230	0.92(0.76-1.1)	0.9(0.69-1.17)	
	60%-80%	12594 (18.6)	342	202	1.14(0.95-1.37)	1.12(0.86-1.45)	
	80%-99%	14439 (21.3)	312	197	1.02(0.84-1.24)	Reference	
	99%-100%	1035 (1.5)	14	11	0.9(0.39-2.06)	0.88(0.37-2.06)	

## Appendix F. Sensitivity Analysis – Live-vaccine risk period analyses at 2- and 4-months to account for possible rotavirus vaccination

**Table:**

Timepoint	TREC Quintile	Vaccinated Children, n(%)	Risk Period Events (Days 4-12)	Control Period Events (Days 20-28)	Relative Incidence (95% CI)	RIR (95% CI)	RIR p-value
2-month	Overall	189715	2724	2689	1.03(0.97-1.09)		
	1	37494 (19.8)	593	549	1.07(0.94-1.21)	1(0.83-1.2)	0.5096
	2	37827 (19.9)	534	554	0.99(0.87-1.12)	0.92(0.77-1.11)	
	3	38108 (20.1)	526	498	1.07(0.94-1.22)	1(0.83-1.21)	
	4	38229 (20.2)	534	560	0.94(0.83-1.08)	0.88(0.73-1.06)	
	5	38057 (20.1)	537	528	1.07(0.94-1.22)	Reference	
4-month	Overall	178356	2322	2377	0.98(0.93-1.04)		
	1	34802 (19.5)	467	438	1.08(0.94-1.25)	1.07(0.88-1.31)	0.5285
	2	35740 (20)	491	509	0.97(0.85-1.11)	0.96(0.79-1.17)	
	3	36157 (20.3)	462	493	0.94(0.82-1.08)	0.93(0.77-1.14)	
	4	35923 (20.1)	454	484	0.92(0.8-1.06)	0.92(0.75-1.12)	
	5	35734 (20)	448	453	1.01(0.87-1.16)	Reference	

## Appendix G. Sensitivity Analysis - including all inpatient and ED events

**Table:**

Confirmed non-live Vaccine Exposure							
Timepoint	TREC Quintile	Vaccinated Children	Risk Period Events (Days 0-2), n	Control Period Events (Days 9-18), n	Relative Incidence (95% CI)	RIR (95% CI)	RIR p-value
2-month	Overall	189715	885	3058	0.93(0.86-1)		
	0-1%	1792 (0.9)	11	36	0.93(0.44-1.97)	0.97(0.44-2.09)	0.59
	1%-20%	35702 (18.8)	150	605	0.81(0.67-0.98)	0.84(0.65-1.09)	
	20%-40%	37827 (19.9)	188	611	1.01(0.85-1.2)	1.04(0.81-1.34)	
	40%-60%	38107 (20.1)	164	606	0.86(0.72-1.03)	0.89(0.69-1.15)	
	60%-80%	38229 (20.2)	189	597	1(0.84-1.19)	1.04(0.81-1.33)	
	80%-99%	36185 (19.1)	171	558	0.96(0.8-1.16)	Reference	
	99%-100%	1873 (1.0)	12	45	0.85(0.43-1.66)	0.88(0.43-1.76)	
4-month	Overall	178356	682	2670	0.82(0.75-0.89)		
	0-1%	1552 (0.9)	12	31	1.11(0.53-2.3)	1.39(0.65-2.96)	0.40
	1%-20%	33250 (18.6)	135	508	0.85(0.7-1.04)	1.07(0.8-1.42)	
	20%-40%	35740 (20)	155	544	0.93(0.77-1.13)	1.17(0.89-1.54)	
	40%-60%	36156 (20.3)	130	521	0.81(0.66-0.99)	1.01(0.76-1.35)	
	60%-80%	35923 (20.1)	124	550	0.69(0.56-0.85)	0.87(0.65-1.16)	
	80%-99%	33952 (19)	121	486	0.8(0.65-0.98)	Reference	
	99%-100%	1783 (1.0)	5	30	0.56(0.21-1.45)	0.7(0.26-1.85)	
6-month	Overall	156903	575	2664	0.68(0.62-0.75)		
	0-1%	1300 (0.8)	8	25	1.09(0.48-2.46)	1.44(0.62-3.32)	0.48
	1%-20%	29428 (18.8)	111	518	0.68(0.55-0.84)	0.9(0.66-1.21)	
	20%-40%	31503 (20.1)	99	531	0.58(0.46-0.73)	0.77(0.56-1.05)	
	40%-60%	31759 (20.2)	130	542	0.75(0.61-0.91)	0.98(0.73-1.32)	
	60%-80%	31532 (20.1)	108	539	0.63(0.51-0.78)	0.83(0.61-1.12)	
	80%-99%	29822 (19)	113	481	0.76(0.61-0.94)	Reference	
	99%-100%	1559 (1.0)	6	28	0.64(0.26-1.56)	0.85(0.34-2.1)	
12-month	Overall	103007	466	2672	0.56(0.51-0.62)		
	0-1%	750 (0.7)	4	24	0.5(0.17-1.45)	0.8(0.27-2.36)	0.78
	1%-20%	18846 (18.3)	72	467	0.5(0.38-0.65)	0.8(0.56-1.12)	
	20%-40%	21433 (20.8)	93	578	0.51(0.4-0.64)	0.81(0.59-1.12)	
	40%-60%	21077 (20.5)	106	560	0.61(0.49-0.76)	0.98(0.71-1.33)	
	60%-80%	20703 (20.1)	93	542	0.57(0.45-0.71)	0.91(0.66-1.25)	
	80%-99%	19179 (18.6)	94	482	0.63(0.49-0.79)	Reference	
	99%-100%	1019 (1.0)	4	19	0.63(0.21-1.86)	1.01(0.33-3.04)	
Confirmed Live attenuated Vaccine Exposure							
Timepoint	TREC Quintile	Vaccinated Children	Risk Period Events (Days 4-12), n	Control Period Events (Days 20-28), n	Relative Incidence (95% CI)	RIR (95% CI)	RIR p-value
12-month	Overall	97998	2825	3058	1.35(1.27-1.43)		
	0-1%	713 (0.7)	23	36	1.75(0.86-3.56)	1.34(0.65-2.77)	0.07
	1%-20%	17951 (18.3)	532	605	1.57(1.36-1.82)	1.21(0.99-1.48)	
	20%-40%	20391 (20.8)	613	611	1.41(1.24-1.61)	1.09(0.9-1.32)	
	40%-60%	20081 (20.5)	552	606	1.15(1.01-1.31)	0.89(0.73-1.07)	
	60%-80%	19636 (20)	563	597	1.36(1.19-1.56)	1.05(0.86-1.27)	
	80%-99%	18222 (18.6)	518	558	1.3(1.13-1.5)	Reference	
	99%-100%	1004 (1.0)	24	45	1.35(0.72-2.54)	1.04(0.54-1.98)	
15-month	Overall	67725	1538	2670	1.12(1.04-1.21)		
	0-1%	438 (0.6)	13	31	1.3(0.57-2.97)	1.09(0.46-2.54)	0.21
	1%-20%	12379 (18.3)	266	508	0.96(0.8-1.14)	0.8(0.62-1.04)	
	20%-40%	14228 (21)	329	544	1.08(0.91-1.28)	0.9(0.7-1.16)	
	40%-60%	13908 (20.5)	291	521	1.06(0.89-1.26)	0.89(0.69-1.14)	
	60%-80%	13639 (20.1)	329	550	1.34(1.12-1.59)	1.12(0.87-1.44)	
	80%-99%	12467 (18.4)	296	486	1.19(0.99-1.43)	Reference	
	99%-100%	666 (1.0)	14	30	1.18(0.52-2.64)	0.99(0.43-2.26)	
